# Strategies for Oligodendrocyte and Myelin Repair in Traumatic CNS Injury

**DOI:** 10.3389/fncel.2020.619707

**Published:** 2021-01-11

**Authors:** Anne Huntemer-Silveira, Nandadevi Patil, Megan A. Brickner, Ann M. Parr

**Affiliations:** ^1^Department of Neuroscience, University of Minnesota, Minneapolis, MN, United States; ^2^Department of Neurosurgery, Stem Cell Institute, University of Minnesota, Minneapolis, MN, United States

**Keywords:** oligodendrocyte, myelin, remyelination, spinal cord injury, traumatic injury

## Abstract

A major consequence of traumatic brain and spinal cord injury is the loss of the myelin sheath, a cholesterol-rich layer of insulation that wraps around axons of the nervous system. In the central nervous system (CNS), myelin is produced and maintained by oligodendrocytes. Damage to the CNS may result in oligodendrocyte cell death and subsequent loss of myelin, which can have serious consequences for functional recovery. Demyelination impairs neuronal function by decelerating signal transmission along the axon and has been implicated in many neurodegenerative diseases. After a traumatic injury, mechanisms of endogenous remyelination in the CNS are limited and often fail, for reasons that remain poorly understood. One area of research focuses on enhancing this endogenous response. Existing techniques include the use of small molecules, RNA interference (RNAi), and monoclonal antibodies that target specific signaling components of myelination for recovery. Cell-based replacement strategies geared towards replenishing oligodendrocytes and their progenitors have been utilized by several groups in the last decade as well. In this review article, we discuss the effects of traumatic injury on oligodendrocytes in the CNS, the lack of endogenous remyelination, translational studies in rodent models promoting remyelination, and finally human clinical studies on remyelination in the CNS after injury.

## Introduction

Oligodendrocytes are the myelin-producing glial cells of the central nervous system (CNS). Myelin plays a critical role in neuronal communication by insulating the axon, enhancing the propagation of action potentials, and facilitating high-frequency firing (Nashmi and Fehlings, 2001; Hartline and Colman, 2007). Besides producing myelin, there is also evidence that oligodendrocytes provide the neurons they ensheath with important trophic support (Garbern et al., 2002; Fünfschilling et al., 2012; Nave and Werner, 2014; Duncan et al., 2020). Disruption of oligodendrocytes or the myelin sheath in injury and disease has severe consequences on neuronal function. Myelin loss decreases cell capacitance and exposes voltage-gated potassium channels, slowing signal transmission and decreasing the likelihood of action potential generation (Nashmi and Fehlings, 2001; Monje, 2018). Additionally, long term effects of oligodendrocyte death beyond demyelination include axonal atrophy and neuronal loss, giving rise to a vicious cycle with axon damage further impacting oligodendrocyte function (Crowe et al., 1997; Nave, 2010). This may result in widespread motor and cognitive deficits and has been identified in several diseases.

Demyelination in the CNS may occur in response to autoimmune disease, genetic mutation, or trauma such as injury or stroke. Multiple sclerosis (MS) is one of the most well studied demyelinating diseases and is characterized by periods of neuroinflammation leading to degradation of myelin in both the gray and white matter of the CNS, with progressive neuronal loss and cognitive impairment in chronic stages (Nave, 2010; Inglese and Petracca, 2015). Leukodystrophies are a family of genetic diseases affecting white matter, a subset of which primarily affects myelin and oligodendrocytes (van der Knaap and Bugiani, 2017). Mutations have been linked to reduced oligodendrocyte differentiation and myelin formation, clinically manifesting in developmental delay, motor dysfunction, seizures, and more (Nave, 2010). Interestingly, substantial research in recent years has implicated myelin abnormality in several psychiatric disorders, highlighting its importance in neuronal transmission from sensorimotor to cognitive tasks (Fields, 2008; Inglese and Petracca, 2015).

Traumatic injury is another major cause of demyelination in the CNS and is the focus of this review article, with an emphasis on brain and spinal cord injuries (TBI and SCI, respectively). Following CNS trauma, damaged axons and oligodendrocytes may trigger demyelination that spreads well beyond the initial injury site, causing impairments in sensory, motor, cognitive, and autonomic function (Alizadeh et al., 2019; Fischer et al., 2020). The Centers for Disease Control and Prevention classify TBI as a serious public health concern, with upwards of 2 million cases per year in the United States alone (Centers for Disease Control and Prevention, 2019). Similarly, there are roughly 20,000 reported cases of SCI annually, with more than 50% resulting in paraplegia or tetraplegia (National Spinal Cord Injury Statistical Center, 2020). The leading causes of both TBI and SCI are preventable accidents such as vehicular collisions and falls (Centers for Disease Control and Prevention, 2019; National Spinal Cord Injury Statistical Center, 2020).

The incidence of demyelination in a variety of disease states makes it an applicable therapeutic target with widespread potential. However, this has also been a subject of controversy based on evidence that complete endogenous remyelination will occur with enough time (Duncan et al., 2020). Definitive reports of chronic demyelination are difficult to ascertain given the complex nature of human injuries and the relatively short time scale of animal models. Endogenous remyelination does occur in both the peripheral and central nervous systems, and in the CNS this mechanism is comparatively less efficient (Franklin and ffrench-Constant, 2008). While time may indeed be a key factor in allowing full remyelination, the consequences of acute demyelination as described above can ultimately lead to cell death before the system can regenerate. Additional processes occurring in tandem during injury and disease may also impair the endogenous response and prevent recovery. Thus, boosting the capacity for remyelination may serve as a way to minimize the long-term damage that follows demyelinating injuries and diseases. In this review article, we will discuss the mechanisms of demyelination and remyelination, with an emphasis on TBI/SCI, followed by an overview of current strategies targeting oligodendrocytes to promote remyelination in different animal models.

## Demyelination Following Traumatic Injury

**Figure 1:**
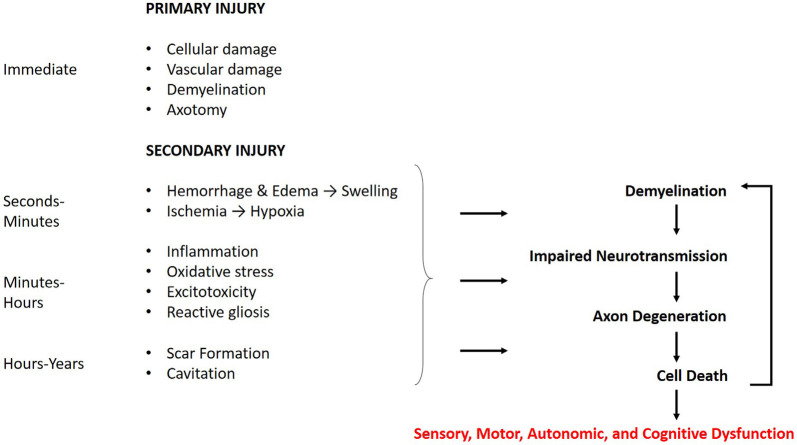
Time course of the cellular response to central nervous system (CNS) trauma. The effects of the primary injury are immediate and directly contribute to secondary damage. The secondary injury begins within minutes of the primary injury and can last for years. The effects of the secondary injury further contribute to the demyelination, axon degeneration, and cell death caused by the primary injury. These mechanisms may manifest in sensory, motor, autonomic, and cognitive dysfunction, with the degree of severity related to that of the injury.

CNS trauma consists of both a primary and secondary injury, with distinct pathophysiology for each (Figure 1). The primary injury involves the direct mechanical impact on the CNS, causing compression, laceration, shearing, etc., and the immediate cellular and vascular damage at the injury site (Johnson et al., 2013; Alizadeh et al., 2019). The primary injury initiates a cellular cascade that gives rise to the secondary injury, the effects of which can last from minutes to years (Crowe et al., 1997; Buss et al., 2004; Ramlackhansingh et al., 2011; Walker and Tesco, 2013). This is distinct from primary and secondary demyelination. Primary demyelination refers to direct injury without axonal compromise, and secondary demyelination occurs indirectly as a result of axonal injury. Both of these can occur as a result of CNS trauma.

Traumatic brain and spinal cord injuries trigger a complex series of secondary events, activating pathways for reactive gliosis, necrosis and/or apoptosis, immune response, and degeneration. Many of the underlying mechanisms for these processes in both TBI and SCI are shared (Bramlett and Dietrich, 2007). Kakulas (2019) provides a clear and detailed description of the secondary cellular events occurring through each stage of SCI in humans. The acute phase occurs immediately after the injury and involves spinal cord swelling, due to hemorrhage and edema, and early necrosis of damaged tissue. The subacute phase begins hours after injury and is characterized by continued necrosis and debris clearing by macrophages. Neuroinflammation and demyelination are also observed. The chronic phase occurs weeks after injury and may persist for years. Phagocytosis of debris leaves behind a cavity at the injury site, and the wall of the lesion contains reactive glial cells that form a glial scar. There is also persisting demyelination and Wallerian degeneration of damaged axons, which progresses both rostral and caudal to the injury. Wallerian degeneration, a hallmark of axonal pathology, involves the catastrophic fragmentation of distal axons as a result of damage and loss of somatic support (Kakulas, 1999, 2019). The same pathologies follow TBI and are described in detail by Bramlett and Dietrich (2007) and Walker and Tesco (2013).

Demyelination has been observed as a result of both primary and secondary injury after TBI/SCI. Traumatic demyelination of damaged axons occurs at the lesion site in primary injury, while demyelination following secondary processes may extend to regions far from the initial insult (Crowe et al., 1997; Kakulas, 1999; Duncan et al., 2020). Slowed impulse conduction as a result of demyelination in the spinal cord contributes to the motor dysfunction and sensory impairment seen in SCI patients (Felts et al., 1997; Nashmi and Fehlings, 2001). Furthermore, axons left without oligodendrocyte support deteriorate over time, subject to extensive Wallerian degeneration, myelin vacuolization, and pathology (Crowe et al., 1997; Oluich et al., 2012). In TBI, demyelination is believed to contribute to the long-term cognitive deficits reported in patients (Walker and Tesco, 2013; Armstrong et al., 2016). In the sections below, we will outline the effects of the primary and secondary injury on oligodendrocytes, the mechanisms behind demyelination and remyelination, and the limitations of endogenous remyelination following CNS trauma.

### Oligodendrocytes and Injury Response

Oligodendrocytes, as generators of myelin, are key to understanding the mechanisms surrounding primary and secondary demyelination in the CNS. The primary injury may directly promote demyelination in two ways. First, the insult itself can strip the myelin from the axons and cause further damage, impairing conduction and downstream signaling. Oligodendrocytes may also be severely damaged from the injury, which can lead to demyelination of the axons they support (Pohl et al., 2011). Importantly, because oligodendrocytes myelinate multiple axons, their loss may affect axons that were spared by the initial injury, causing progressive or chronic demyelination extending beyond the lesion site (Conti et al., 1998; Emery et al., 1998; Buss et al., 2004). Myriad additional secondary processes provoke demyelination as well, however, the complex nature of the secondary injury poses an obstacle to discerning the relationship between oligodendrocytes, demyelination, and axonal degeneration.

The mechanisms of secondary injury are complex and have widespread effects. As such, some degree of compartmentalization is necessary for understanding the responses of different cell types. Previous work has uncovered several aspects of the lesion environment that increase the sensitivity and vulnerability of oligodendrocytes following injury. For example, excessive neurotransmitter release from necrotic tissue may cause glutamate and ATP mediated excitotoxicity in surrounding cells. Oligodendrocytes appear quite susceptible to this and often undergo excitotoxic cell death after injury (Xu et al., 2004; Butt, 2006; Domercq et al., 2010). Proteolytic enzymes released during necrosis may further potentiate damage. Free radical formation due to ischemia contributes to oxidative stress, to which oligodendrocytes demonstrate increased vulnerability (Thorburne and Juurlink, 1996; Giacci and Fitzgerald, 2018). The invasion of immune cells, release of inflammatory cytokines by microglia, and infiltrating neutrophils have also been associated with oligodendrocyte loss (Donnelly and Popovich, 2008; Satzer et al., 2015). These factors in turn increase the numbers of necrotic, apoptotic, and autophagic oligodendrocytes after CNS trauma (Kanno et al., 2009; Almad et al., 2011). It should be noted, however, that these responses are complex and serve both protective and harmful roles (Jones et al., 2005; Kotter et al., 2005; Ohri et al., 2018). Other cell types, such as neurons and astrocytes, also demonstrate varying degrees of sensitivity to these processes, and thus care should be taken to note the differential effects of targeting these mechanisms experimentally.

Apoptosis of oligodendrocytes has been reported in several TBI/SCI studies (Crowe et al., 1997; Conti et al., 1998; Emery et al., 1998; Lotocki et al., 2011; Flygt et al., 2013). As mentioned above, oligodendrocyte death may initiate demyelination in associated axons. However, axonal degeneration also appears to trigger oligodendrocyte apoptosis, as reported in Crowe et al. (1997) where Wallerian degeneration preceded the emergence of adjacent apoptotic CC1/RIP positive cells. This corroborates other work demonstrating a dependence of oligodendrocytes on neuronal activity for survival and highlights the reciprocal quality of their relationship (Barres et al., 1993). A chain effect thus emerges, whereby damaged axons trigger apoptosis in oligodendrocytes, causing further demyelination of distal axons. This intricate relationship sheds light on the motivation behind utilizing oligodendrocyte replacement and myelin growth-promoting factors after CNS injury to prevent widespread deterioration. Multiple studies utilizing such approaches in acute and subacute models of SCI show promising evidence of functional recovery (Hwang et al., 2009; Erceg et al., 2010; Kawabata et al., 2016). Similar improvements in chronic SCI models have been more difficult to achieve, possibly because this progressive demyelination and degeneration have gone unchecked for much longer (Keirstead et al., 2005; Karimi-Abdolrezaee et al., 2006; Nishimura et al., 2013).

### Mechanisms of Remyelination After Injury

The remyelination of axons following injury in many ways resembles the myelination of the developing CNS, with some key temporal and molecular differences (Gallo and Deneen, 2014; Almeida, 2018). Much of the attention on remyelination in the CNS has focused on oligodendrocyte progenitor cells (OPCs). Franklin and ffrench-Constant (2017) describe the three phases of OPC mediated remyelination in the CNS: activation, recruitment, and differentiation. During activation, OPCs undergo a change in gene expression allowing them to become proliferative (Moyon et al., 2015). During recruitment, OPCs originating both locally and from the subventricular zone (SVZ) migrate to the lesion site in response to injury (McTigue et al., 2001; Caillava et al., 2011; Flygt et al., 2013; Xing et al., 2014). Finally, OPCs exit the cell cycle and differentiate into myelin-producing oligodendrocytes. These processes involve a diverse array of mediators and molecules, varied by regional cellular context, that guide remyelination (Brosius-Lutz and Barres, 2014; Gallo and Deneen, 2014; Almeida, 2018). Pharmacological manipulation of identified receptors and signals is a common approach for promoting remyelination and will be discussed in more detail in later sections.

The capacity for mature oligodendrocytes to contribute to remyelination is a topic of debate. It has long been held and supported that because oligodendrocytes are post-mitotic and differentiated, they are both unable to migrate and also fail to remyelinate after damage (Blakemore and Keirstead, 1999; Crawford et al., 2016; Pukos et al., 2019). Indeed, it seems likely that OPCs provide the majority of remyelination after injury, as OPC transplantation greatly improves measures of myelination (Groves et al., 1993; Keirstead et al., 2005). However, recent reports suggest that mature, intact oligodendrocytes do engage in remyelination following injury (Yeung et al., 2014; Jeffries et al., 2016; Duncan I. D. et al., 2018; Macchi et al., 2020). The variability in findings likely relates to differences in cell lines, animal models, the type of demyelinating injury, and methods of evaluating myelination across studies. Additional work emphasizing consistency across approaches will be necessary to determine the validity of promoting oligodendrocyte vs. OPC survival as a strategy following CNS trauma.

The process of myelination itself is also quite complex. The successive wrapping of myelin layers is driven by actin dynamics and requires interactions between several adhesion molecules, actin-binding proteins, and, critically, myelin basic protein (Nawaz et al., 2015; Zuchero et al., 2015; Klingseisen et al., 2019). There is an activity-dependent component of myelination as well. Not only do OPCs preferentially activate in regions with elevated electrical activity, but oligodendrocytes themselves demonstrate a predilection to myelinate more active axons (Barres and Raff, 1993; Gibson et al., 2014; Hines et al., 2015). Though this is believed to underlie the specificity of myelination *in vivo*, the mechanisms behind it are unclear. Several growth factors and signaling molecules have been implicated thus far (Ronzano et al., 2020). For example, the trophic factor BDNF, secreted by neurons, astrocytes, oligodendrocytes, and Schwann cells, modulates myelination in an activity-dependent manner (McTigue et al., 1998; Ikeda et al., 2002; Lundgaard et al., 2013). This may have ramifications for the use of electrical stimulation as a treatment for peripheral nerve regeneration and SCI. For both of these pathologies, electrical stimulation has been shown to increase BDNF levels (McGregor and English, 2019; Ghorbani et al., 2020). Thus, BDNF-mediated remyelination may underlie some of the functional recovery seen in SCI patients receiving epidural stimulation (Pettigrew et al., 2017).

Schwann cell-mediated remyelination can also occur following SCI. Schwann cells, which are the myelinating cells of the peripheral nervous system, are known to infiltrate the spinal cord after SCI and in other demyelinating diseases (Duncan and Hoffman, 1997). Whether this process is beneficial or detrimental is also a subject of some controversy. It should be noted that, unlike oligodendrocytes, Schwann cells typically myelinate in a 1:1 ratio, making them potentially less efficient in comparison. However, Schwann cells have been used in transplantation studies to promote remyelination, and grafts of Schwann cells improve regeneration of CNS axons (Aguayo et al., 1981; Kanno et al., 2015). Furthermore, Schwann cells are being actively pursued in clinical trials for use in SCI, with phase I testing complete (Anderson et al., 2017).

Finally, it is important to note the extent to which remyelination alone contributes to functional recovery following TBI/SCI is not clearly discerned (Myers et al., 2016; Duncan G. J. et al., 2018). This lack of clarity arises because oligodendrocytes provide other forms of support to surrounding neurons, such as secretion of growth factors and immune modulators (Du and Dreyfus, 2002; Assinck et al., 2017). Oligodendrocytes may then be promoting recovery through other mechanisms of action besides or in addition to remyelination. This has been proposed as an alternate explanation for why recovery of function is seen in acute and subacute SCI but not chronic models. Furthermore, the early recovery pattern observed in some of these studies suggests that at least some functional recovery occurs before remyelination (Duncan G. J. et al., 2018). This is not to suggest that remyelination is an inadequate target for repair strategies; rather it emphasizes the need for combined approaches that can act on multiple aspects of the repair process to achieve the best outcomes.

### Barriers to Endogenous Remyelination

**Figure 2:**
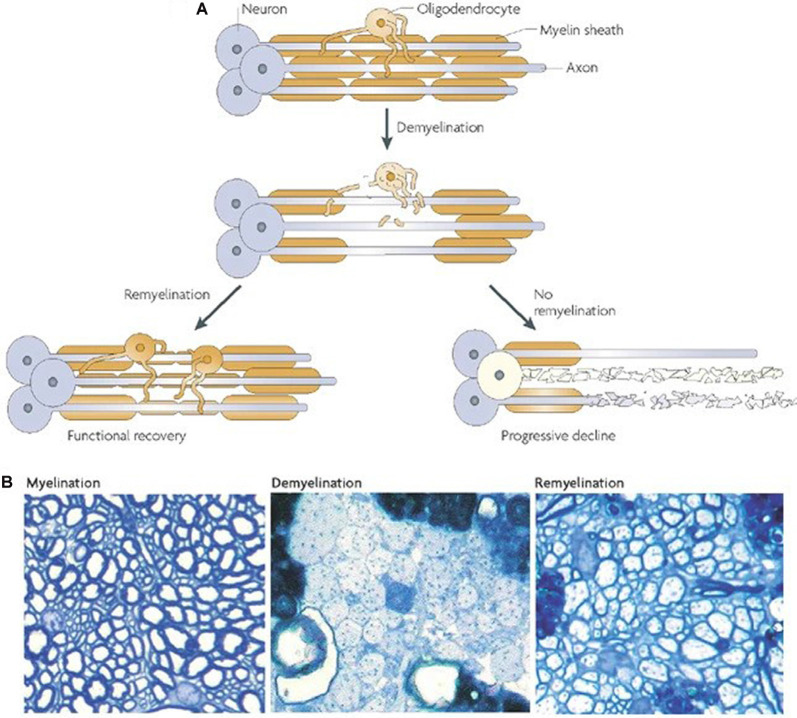
Outcomes of demyelination. **(A)** A demyelinated axon will either be remyelinated, giving at least partial restoration of function, or it will undergo Wallerian degeneration. **(B)** Transverse sections from adult rat cerebellar white matter, fixed in resin and imaged with light microscopy. The left panel depicts normally myelinated axons. The middle panel shows demyelinated axons following injection with ethidium bromide. The right panel shows remyelinated axons 4 weeks after ethidium bromide injection. It should be noted that the new myelin is thinner and shorter (**A,B** from Franklin and ffrench-Constant, 2008).

Endogenous remyelination in the CNS is limited and often fails to provide functional recovery (Franklin and ffrench-Constant, 2008; Boyd et al., 2013; Franklin and Goldman, 2015). The reasons for this are intricate and many components are poorly understood. Figure 2 depicts the possible outcomes following demyelination. Evidence suggests that when the axon is preserved remyelination occurs naturally and may restore both signal conduction and sensorimotor function (Smith et al., 1979; Duncan et al., 2009). However, the newly formed myelin is thinner and conduction amplitude is lower than in normally myelinated axons, implying an intrinsic limitation in the capacity for remyelination (Smith et al., 1979; Nashmi and Fehlings, 2001; Franklin and ffrench-Constant, 2017). Furthermore, the Wallerian degeneration of damaged axons poses a major obstacle to remyelination. This loss of axons and the limited capacity of the CNS to regenerate hinders prospects for remyelination. Indeed, one study utilizing a demyelinating model of MS found that remyelination alone was not sufficient for sustained functional recovery when axonal degeneration persisted (Manrique-Hoyos et al., 2012). As such, therapies focused on remyelination must also consider pairing with strategies to boost axon growth.

As discussed previously, adult precursor cells occupying the CNS respond to demyelinating injuries. However, compared to other species, the contribution of these cells to mammalian regeneration is attenuated. There are three well-established barriers to axon regeneration in the CNS: the glial scar, myelin inhibitory molecules, and lack of trophic support. Though evidence exists in support of all of these factors, the extent to which each contributes overall to regeneration failure remains to be determined. For this review article, they will be discussed briefly.

The glial scar has been a subject of controversy for years, with conflicting claims demonstrating both protective and detrimental effects to recovery following injury. The scar is the result of reactive gliosis extending into the chronic phase of trauma, leaving behind a dense layer of astrocytes surrounding the lesion cavity. First, this acts as a thick physical barrier, preventing axons that may otherwise regenerate from crossing the cavity. The extracellular matrix of the glial scar is also rich in chondroitin sulfate proteoglycans (CSPGs), some subtypes of which are inhibitory to axon growth (Mckeon et al., 1995; Barritt et al., 2006; Silver, 2016). On the other hand, CSPGs that stimulate axon growth and survival have also been identified in the glial scar, lending to arguments of its multifaceted function (Nichol et al., 1994; Nakanishi et al., 2006; Schäfer and Tegeder, 2018). This is further complicated by studies showing little to no effect of glial scar ablation on regeneration (Anderson et al., 2016; Patil et al., 2018; Haindl et al., 2019). Together, the current literature suggests that the glial scar initially serves to prevent healthy axons from entering a region of inflammation and necrosis. Once the debris from the injury has been cleared, however, its presence may impede regeneration and recovery (Rolls et al., 2009). It remains uncertain whether the glial scar affects oligodendrocyte migration and remyelination as well.

A major breakthrough in the study of axon regeneration came from the discovery of molecules within the myelin itself that inhibit axon growth (Caroni and Schwab, 1988). Many such molecules have now been identified. Among the most characterized are Nogo-A, myelin-associated glycoprotein (MAG), and oligodendrocyte myelin glycoprotein (OMgp; McKerracher et al., 1994; Kottis et al., 2002; Huebner and Strittmatter, 2009). These three molecules converge on the shared Nogo receptor (NgR1), which interacts with the transmembrane protein LINGO-1 to limit axon growth (Mi et al., 2004). While these molecules are enriched in CNS myelin, they are largely absent from PNS myelin. This is believed to contribute to the differences in the regeneration capacity of CNS and PNS axons (Huebner and Strittmatter, 2009). Further research is needed in this regard, as studies utilizing animal knockouts for these molecules show mixed results (Bartsch et al., 1995; Wong et al., 2003; Lee et al., 2010). This will be critical for the development of effective combination therapies, as remyelination strategies may be more beneficial if applied after treatments for neuronal regeneration. The optimal temporal relationship between regeneration and remyelination requires further elucidation.

CNS axons also receive diminished trophic support after injury compared to the peripheral nervous system. Following injury, Schwann cells in the PNS upregulate numerous neurotrophins and transcription factors, and impairing this process significantly increases cell death (Arthur-Farraj et al., 2012; Brosius-Lutz and Barres, 2014). Also, Schwann cells provide structural support to healthy, uninjured axons through the secretion of the basal lamina, which is prevalent throughout the PNS. The presence of the basal lamina is critical following peripheral nerve injury, as it provides a laminin-rich substrate for axon regrowth (Chen et al., 2007). Oligodendrocytes, on the other hand, do not supply equivalent aid in the CNS, and this is believed to account for some differences in regeneration between the CNS and PNS. Several therapeutic strategies have harnessed the use of neurotrophins and Schwann cell transplantation in models of CNS trauma to enhance regeneration (Aguayo et al., 1981; Kelamangalath and Smith, 2013; Kanno et al., 2015).

For both remyelination and axon regeneration, age is an unavoidable risk factor that diminishes the body’s endogenous response (Sim et al., 2002; Franklin and ffrench-Constant, 2008; Hampton et al., 2012). This intrinsic decrease in capacity over time poses a major hurdle for clinical treatment, as falls in the elderly make up a substantial population of CNS trauma cases (Centers for Disease Control and Prevention, 2019).

While these barriers to remyelination and regeneration have been a source of consternation for scientists and clinicians alike, technological advances in the last decade have greatly improved prospects. One preprint from the Blackmore lab utilizing cell transplantation paired with pro-regenerative gene delivery and growth permissive grafting showed enhanced axon regeneration and functional recovery in a murine SCI model (Jayaprakash et al., 2019). Such combination therapies hold considerable promise in the treatment of TBI/SCI.

## Translational Approaches in Animal Models

Remyelinating therapies help restore metabolic support to degenerating axons and facilitate the restoration of proper conduction and function. Most available treatments target the prevention of progressive demyelination that contributes to chronic disability, but few can promote myelin repair. This section will discuss the translational methodologies utilized in animal models that have made progress towards clinical application.

Several species and models that have contributed to the study of demyelination in injury and disease will be highlighted below. Available models for traumatic injury range in type and severity, lending important insights into the capacity of the CNS for recovery based on the degree of damage. The most common TBI models include fluid percussion, weight drop, and cortical contusion injuries, which may be focal or diffuse (Albert-Weissenberger and Sirén, 2010). Similarly, SCI models range from mild compression or contusion to complete transection (Kjell and Olson, 2016). Rodent models are most commonly used for TBI/SCI, though large animal models are also utilized and are critical for moving toward clinical application. In addition to models of demyelinating diseases have not only contributed to the current understanding of demyelination in general but have also greatly informed the field of CNS trauma. The *shiverer* mouse model, possessing a mutation in the gene for myelin basic protein, is among the most common of these and is often used to study leukodystrophies (Nave, 2010). The Long Evans shaker rat model also has a mutation in the myelin basic protein gene, resulting in widespread loss of myelin (Kwiecien et al., 1998). Other demyelination models rely on the injection of toxins, such as ethidium bromide or lysolecithin, followed by irradiation (Kulbatski et al., 2008). Findings from each of these models will be included in the sections that follow, and for simplicity will be referred to as either “TBI/SCI models” or “demyelination models.”

### Small Molecules

Remyelination is governed by several extrinsic and intrinsic factors that act either as inhibitors or stimulators of OPC differentiation (Kremer et al., 2016; Lopez Juarez et al., 2016; Gruchot et al., 2019). As discussed above, following demyelinating damage, endogenous OPCs from the SVZ migrate to the demyelinated area and differentiate into mature myelinating oligodendrocytes to aid in remyelination. This has been shown in several animal models of demyelination (Nait-Oumesmar et al., 2007; Kim and Szele, 2008; Mecha et al., 2013). However, remyelination mediated by this process is inefficient and ultimately incomplete in several disease states, such as MS, due in part to inadequate OPC differentiation. Small molecules can be used to enhance this endogenous process. Göttle et al. (2019) classified the small molecules that aid in remyelination into three major groups: (1) receptor/membrane-bound molecules; (2) physiologically occurring free molecules; (3) non-physiologically occurring free molecules. Here we will discuss the use of these small molecules as a therapeutic strategy to advance remyelination in pathologically demyelinated axons in the CNS.

Many receptor systems activate pathways that control myelination. G-protein coupled receptors (GPCRs) are among the most ubiquitous in this regard and thus offer a promising therapeutic target. Two specific GPCRs identified for their role in myelination are the muscarinic receptor 1 (M1R) and the kappa opioid receptor (KOR). Researchers have employed various strategies to develop small molecules that can target these pathways. One approach is to repurpose the US Food and Drug Administration (FDA) approved drugs such as benzatropine (anticholinergic), clemastine (antihistamine), and miconazole (antifungal). These show potential in enhancing oligodendrocyte formation *in vitro* and promoting functional remyelination in animal models of MS (Deshmukh et al., 2013; Najm et al., 2015; Hubler et al., 2018). Similarly, Mei et al. (2014, 2016a) identified a cluster of antimuscarinic molecules, which includes eight FDA-approved compounds, that enhance oligodendrocyte differentiation and remyelination. This group also identified a cluster of KOR agonists that significantly promotes oligodendrocyte differentiation and myelination in mice (Mei et al., 2016b). Furthermore, clemastine, a small molecule that stimulates significant OPC differentiation *in vitro* through the M1 muscarinic receptor, provides drug-induced repair and remyelination in multiple animal models (Li et al., 2015; Liu et al., 2016; Mei et al., 2016a; Zada et al., 2016). Clemastine is now available as an over-the-counter antihistamine that is being developed as a potential treatment for relapsing-remitting MS and has advanced into clinical trials (Green et al., 2017). Another family of GPCRs that has received increasing attention is the GPR17 receptors, which act as sensors of local damage to the myelin sheath. Modification of GPR17 activity promotes oligodendroglial maturation *in vitro* and in animal models (Merten et al., 2018; Dziedzic et al., 2020; Parravicini et al., 2020). Finally, the protective effects of pregnancy on MS relapse have led to the extensive focus on the estrogen receptor (ER) and its ligands as a means for promoting remyelination (Xiao et al., 2012; Moore et al., 2014; Najm et al., 2015; Itoh et al., 2017). Of particular note is the small molecule NDC1308, an estrogen receptor agonist that has been proposed as a remyelination therapy for several demyelinating and neurodegenerative diseases (Nye and Yarger, 2017). Whether these treatments can be applied to CNS trauma has yet to be tested.

The targeting of endogenous small molecules to promote remyelination specifically in CNS trauma has yielded some promising results. For instance, the RhoA family of GTPases are key regulators of neurite outgrowth, axon regeneration, and OPC migration. Acting on this pathway, the Vav family of GEFs poses a potential therapeutic target to improve and speed myelin repair (Ulc et al., 2017). Rac and Cdc-42 can also be used to promote OPC development, as they act in opposition to RhoA (Liang et al., 2004). Small molecules inhibiting Notch and Nogo-A signaling promote oligodendrocyte and myelin formation (Huebner and Strittmatter, 2009; Franklin and ffrench-Constant, 2017; Göttle et al., 2019). Also, Hubler et al. (2018) describe several pro-myelinating small molecules that function by directly inhibiting enzymes in the cholesterol biosynthesis pathway. The subsequent accumulation of the substrates of these enzymes promotes oligodendrocyte formation. These findings demonstrate the utility of enhancing the formation of oligodendrocytes as a target for the development of optimal remyelinating therapeutics.

In both traumatic brain and spinal cord injury, small molecules have been used to enhance the proliferation of endogenous stem cells. Multipotent stem cells from the SVZ, adjacent to the ventricles of the brain and central canal of the spinal cord, contribute to the formation of new oligodendrocytes following injury (Morshead and van der Kooy, 1990). Although early reports in amphibians and urodeles suggested these cells contribute to robust regeneration, in mammals this response is attenuated and does not produce significant recovery (Zhao et al., 2016). The use of growth factors such as FGF and EGF has been shown to stimulate this proliferative response and possibly improve physiological recovery in rodent models of SCI (Kojima and Tator, 2002; Parr and Tator, 2007).

A key difficulty in utilizing small molecules is determining the time point at which administration will be most beneficial. While many endogenous molecules have been implicated in remyelination, each pathway acts on different components of the process and thus requires specific temporal specification. Proper application of small molecule treatment following these temporal features ensures efficacy and will be especially important when applied in conjunction with other therapies.

Small molecules currently in ongoing clinical trials include Quetiapine, a nonselective GPCR antagonist, GSK239512, a histamine H3 receptor antagonist, Domperidone, a D2/D3 dopamine receptor antagonist, and Olesoxime, a cholesterol-like compound (Bothwell, 2017). In early 2020 Sanofi conducted a phase 2b study evaluating a BTK inhibitor, SAR442168, which reduced disease activity associated with MS (Sanofi: Press Releases, 2020). Genentech also initiated a Phase III clinical trial for fenebrutinib, another BTK inhibitor found to be effective against multiple types of MS (Genentech: Press Releases, 2020).

### Monoclonal Antibodies

Monoclonal antibodies (MAbs) refer to antibodies produced *in vitro* by a single clone of identical cells. Originally MAbs were used in research to study the mechanisms of antibody specificity and to generate reagents for pathogens (Lu et al., 2020). MAbs are now also utilized as therapeutic strategies for many disease conditions (Wang, 2011). Advancements have been particularly potent for cancer and autoimmune disorder therapies as well as the containment of infectious diseases (Walker et al., 2009; Lu et al., 2020). Recently, their application has expanded to that of CNS trauma.

While the literature on the use of MAbs specifically for remyelination post-injury is limited, there have been numerous studies that resulted in MAb-induced axonal regeneration and remyelination. Inflammation, which contributes to the widespread necrosis and apoptosis seen following injury, may be a valuable target for remyelination. For example, the inflammatory cytokine IL-20 is upregulated in the glial cells of rats after SCI and may play a role in the inhibition of axonal regeneration (Dumoutier et al., 2001). *In vivo* treatment of an anti-IL-20 MAb, 7E, following SCI in rats suppressed inflammatory responses, preserved myelin, reduced glial scar formation, and provoked improved motor and sensory function (Lee et al., 2020). Leukocytes, which release proinflammatory cytokines and free radicals that damage white and gray matter, are another potential target for remyelination (Blight, 1985; Carlson et al., 1998; Taoka and Okajima, 1998). Leukocytes are carried by the blood *via* interactions with endothelial cell adhesion molecules, and administration of antibodies blocking the CD11d/CD18 integrin involved in this process have improved motor function, preserved myelin, and reduced secondary damage following SCI (Gris et al., 2004; Hurtado et al., 2012).

MAbs have also been used to target neuroinflammation in TBI (Lenzlinger et al., 2001; Unterberg et al., 2004). Treatment with the anti-CD11d integrin MAb described above reduces levels of leukocyte infiltration, lessens neuroinflammation and neuronal loss, and is associated with increased behavioral functioning in multiple models of TBI (Utagawa et al., 2008; Bao et al., 2012; Shultz et al., 2013). High mobility group box-1 (HMGB1) protein is another molecule that mediates inflammation following injury (Scaffidi et al., 2002). An anti-HMGB1 MAb hindered the protein’s translocation, reduced expression levels of inflammatory molecules, and improved motor function following a fluid percussion TBI (Okuma et al., 2012). The proinflammatory lysophosphatidic acid (LPA) is also upregulated after TBI in humans and mice (Frugier et al., 2011). Inhibiting LPA signaling *via* administration of B3, an anti-LPA MAb, following an injury can reduce lesion size and expression levels of inflammatory cytokines, as well as improve motor deficits (Crack et al., 2014). After mouse SCI, application of B3 suppresses the formation of a glial scar and enhances neuronal survival, neurite sprouting, and motor function (Goldshmit et al., 2012).

Treatments targeting growth cone function through the RhoA pathway have also been successful in promoting remyelination following CNS trauma. The IN-1 antibody, which neutralizes neurite growth inhibitors, has been utilized to infiltrate this signaling pathway (Mohammed et al., 2020). IN-1 MAb supports axonal regeneration following SCI (Schnell and Schwab, 1990; Brösamle et al., 2000). Another promising target is the repulsive guidance molecule A (RGMa), which accumulates in lesion sites of SCI (Schwab et al., 2005; Hata et al., 2006). RGMa is a protein-ligand that binds to the Neogenin receptor and is ultimately involved in neurite growth inhibition (Tassew et al., 2014). This mechanism depends on Rho kinase pathway activation. After SCI, RGMa can be neutralized by rat antibodies, which subsequently promotes axon regeneration and locomotor improvement (Hata et al., 2006). Administration of human anti-RGMa MAbs after rat SCI promotes neurobehavioral functioning, decreases neuronal apoptosis, and induces corticospinal tract axonal growth (Mothe et al., 2017, 2020). Induction of CST axonal sprouting and functional recovery has also been reported in primates (Nakagawa et al., 2019).

Similarly, MAbs for the myelin inhibitory molecules discussed earlier have shown improvement in promoting remyelination. Recent studies investigated combination treatments involving anti-Nogo-A MAbs in rat SCI. Combination therapy with methylprednisolone (MP) produced elevated axonal remyelination levels and greater locomotor function (Wu et al., 2014). Furthermore, an anti-MAG MAb treatment in a rat fluid percussion TBI model preserved hemispheric tissue volume and neuromotor function (Thompson et al., 2006).

The use of MAbs specifically for remyelination is better characterized in animal models of MS but may have translational potential to TBI/SCI as both result in demyelination. The main focus has been on the oligodendrocyte-reactive IgMκ subclass of MAbs because antibodies that do not bind to oligodendrocytes will not induce remyelination (Asakura et al., 1998; Bieber et al., 2002). Investigations of multiple IgMκ MAbs in Theiler’s murine encephalomyelitis virus (TMEV) model of MS indicates they may prompt remyelination by directly binding to damaged oligodendrocytes, which initiates an immune effector mechanism (Asakura et al., 1998). Human IgM MAbs advanced remyelination efforts in the TMEV model at equal to higher degrees than the polyclonal human IgM (Warrington et al., 2000). Mice treated with a human serum MAb, sHIgM22, demonstrated an increase of axonal remyelination after CNS demyelination. Interestingly, the sHIgM22 treatment condition was associated with high levels of lesion adjacent macrophages. This suggests a potential role of antibody-induced macrophage recruitment in remyelination (Bieber et al., 2002). The recombinant form of human IgM MAb, rHIgM22, can also stimulate remyelination after TMEV-induced myelin loss in mice (Warrington et al., 2001). Assessment of TMEV infected mice before and after rHIgM22 treatment confirmed a decrease in spinal cord lesion tissue volume, mediated by MAb-induced remyelination within the spinal cord (Pirko et al., 2004). The majority of lesions associated with TMEV are found in the spinal cord, which may provide translatability to SCI models and remyelination efforts. The evidence of IgM MAb-induced remyelination in models of MS provides a starting point for the potential efficacious treatment of TBI/SCI, especially for remyelination.

### RNA Interference

RNA interference (RNAi) is both a physiological process and a therapeutic technique using microRNA (miRNA), short hairpin RNA (shRNA), and small interfering RNA (siRNA) to silence genes in a sequence-specific manner. Its development revolutionized gene therapy and has grown in the last few years, with two commercially available disease-targeting siRNA therapeutics approved since 2018 (Hu et al., 2020). Many more clinical and preclinical trials are in the works (Scherman et al., 2017). RNAi molecules bind the mRNA sequence of specific genes and cleave them, preventing translation into functional proteins. In contrast to small molecules and monoclonal antibodies, which target active proteins and pathways, RNAi interacts with the mRNA, allowing for greater specificity. Because it pairs with the sequence itself, this approach is ideal for diseases where a high-affinity molecule for a protein or receptor of interest has not been identified.

The research and development of RNAi have overcome many hurdles to achieve medical application. First, naked siRNAs are both unstable and subject to rapid degradation by nucleases, rendering them ineffective. Second, off-target effects are also possible, though rare, and physiological responses vary widely based on the tissue the drug is administered to. These concerns have been minimized by the production of chemically modified siRNAs, increasing their strength and precision. Additionally, numerous viral and nonviral delivery methods for RNAi have been refined, allowing for application to many types of organs and tissues (Grimm and Kay, 2007; Hu et al., 2020).

As of now, most treatments utilizing RNAi focus on heritable and neurodegenerative diseases, with little work in the realm of CNS trauma. However, these investigations are clearly translatable to the field of TBI/SCI. One example is the use of RNAi to manipulate cell death pathways for tumor suppression (Crnkovic-Mertens et al., 2003; Uchida et al., 2004; Kock et al., 2007). These successes in cancer research offer an encouraging way to minimize the widespread apoptosis seen for months following traumatic injury (Rink et al., 1995; Emery et al., 1998). Preliminary results using a cocktail of siRNAs to silence pro-apoptotic proteins following SCI showed evidence of improved myelination and decreased cell death (Michael et al., 2019). Further evidence comes from Niu et al. (2012) who used siRNA targeting the Wnt/Ca^2+^ pathway to prevent the pathological accumulation of calcium following TBI.

There is a small but growing pool of literature using RNAi in animal models of SCI and demyelinating disease that has shown promise. Studies employing siRNA to silence the expression of myelin inhibitory molecules, such as NoGo-A, demonstrate functional recovery in murine models of multiple sclerosis and SCI (Yang et al., 2010; Sun et al., 2012). Other laboratories have instead targeted cytoskeletal proteins and signaling molecules to improve motility and regeneration after SCI (Yu et al., 2010; Qu et al., 2014; Ding et al., 2017). Similarly, there are several secondary cascades affecting oligodendrocytes and remyelination after injury that may be feasible targets for RNAi. For instance, minimizing oxidative stress following SCI may decrease the vulnerability of neurons and oligodendrocytes to the lesion environment, lessening cell death and demyelination. A study by Gao and Li (2017) successfully silenced inducible nitric oxide synthase (iNOS) expression in M1 macrophages following acute SCI in rats, correlating with both a substantial decrease in the expression of apoptotic markers and an increase in anti-apoptotic markers. Functional testing was not performed in this experiment, but a similar study silencing IRF5 in macrophages in a mouse model of SCI found significant motor recovery as well as decreases in demyelination and inflammation (Li et al., 2016). Another experiment silencing nitric oxide synthase in a TBI model showed decreased neuronal degeneration in addition to improved working memory (Boone et al., 2017). As of now, most experiments using RNAi in TBI models focus on minimizing post-injury edema (Fukuda and Badaut, 2013; Xu et al., 2014; Guan et al., 2020).

While these findings have prompted excitement for the future of RNAi as a therapy for traumatic injury, several concerns must be kept in mind moving forward. First, the heterogeneous nature of the brain and spinal cord could result in differential uptake of siRNAs by different cell types with unintended consequences (Michael et al., 2019). However, the use of antibody-tagged siRNAs has been shown to increase the specificity of uptake to certain cell types (Liu, 2007). It is also important to note the key differences between experimental and clinical CNS injuries. Scientists take great precautions to ensure injuries are precise and consistent between animals. Human TBI/SCI cases, on the other hand, are each unique, with a great deal of variability from person to person. For developing translatable RNAi therapeutics, it is thus critical that treatments can be tailored to the individual to ensure the best outcomes.

### Cell Replacement Strategies

**Figure 3:**
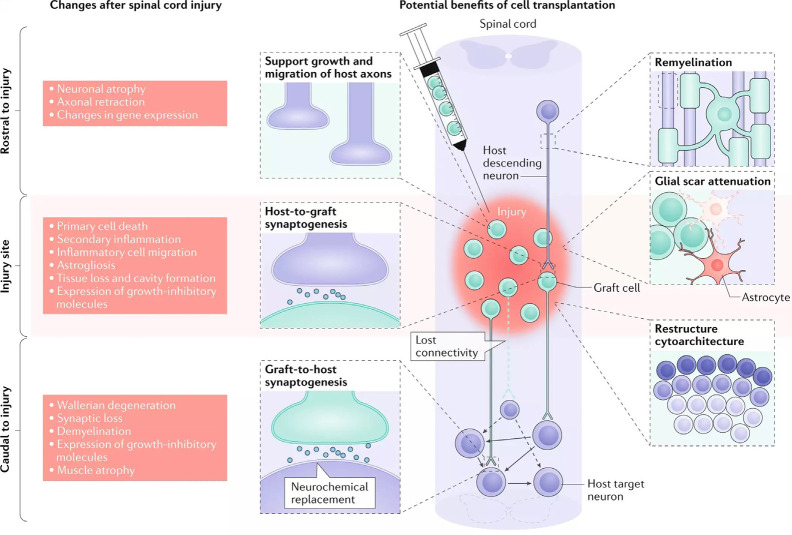
Cellular response to spinal cord injury and the possible therapeutic benefits of cell replacement therapy (CRT). The left depicts the changes that occur in the spinal cord following injury, both rostral and caudal to the lesion site. The right details targeted outcomes of cell transplantation, including remyelination, synaptogenesis, axon regeneration, glial scar attenuation, and the restructuring of cytoarchitecture (adapted from Fischer et al., 2020).

Oligodendrocytes and OPCs are critical for promoting remyelination of the injured CNS. However, the death of oligodendrocytes and the failure of OPCs to adequately replenish them contributes significantly to the persistent neuronal demyelination and degeneration seen in trauma and disease (Franklin and ffrench-Constant, 2017). As a result, cell replacement therapy (CRT) offers an encouraging means of restoring oligodendrocyte and progenitor populations to improve remyelination (Figure 3). Both neural and non-neural cells have been used in CRT, though we will be focusing on neural cells and their potential to directly restore myelin. Additional benefits of CRT include neuroprotection, provision of regenerative molecules, modulated immune response, and neurological recovery (Assinck et al., 2017; Fischer et al., 2020). CRT has been tested in both injury and disease models with varying degrees of success.

Advancements in stem cell biology have greatly contributed to the prospects of CRT as a viable clinical treatment. Human pluripotent stem cells provide a highly responsive and programmable pool that can be differentiated into numerous cell types. Research to date has utilized stem cells obtained from several sources: fetal stem cells, embryonic stem cells (ESCs), induced pluripotent stem cells (iPSCs), and neural stem cells (NSCs) derived from the adult nervous system. There is further diversity with regards to the differentiation protocols themselves as well as the stage at which these cells are transplanted. Cell transplantation protocols using NSCs, OPCs, mature oligodendrocytes, and stages in-between have all been documented to improve remyelination. While this demonstrates utility in stem cell application, there is now a need for a consolidation of methodologies as we move towards clinical translation.

An eventual goal of CRT is autologous transplantation, in which the patient’s own cells are used to supplement the site of demyelination, however, many steps remain before this can be achieved. Early work laid the foundations towards this goal through the culture and transplantation of rodent ESCs into the rodent spinal cord (McDonald et al., 1999; Liu et al., 2000; Karimi-Abdolrezaee et al., 2006). These studies also reported increased myelination in the hosts, indicating at least some ES cells differentiated into myelinating oligodendrocytes after transplantation. Subsequent efforts have now shifted towards the transplantation of human stem cells into rodent models. Although this comes with the added difficulty of requiring substantial immune suppression of the animals to prevent rejection of the transplants, it also gives invaluable information regarding the safety of transplanting these cells (Forsberg and Hovatta, 2012).

Some of the earliest differentiation protocols direct cells toward a neural fate, allowing for the differentiation of different neural cell types at different stages (Cummings et al., 2005; Parr et al., 2007; Nishimura et al., 2013). Here, cells are considered NSCs or neural progenitor cells (NPCs), terms which have been used somewhat interchangeably in the literature. There are several benefits to transplanting at this early phase. First, because these cells are still highly responsive to exogenous signals, their differentiation can be tailored to the environment of the host nervous system. For instance, the increased expression of factors signaling the recruitment and differentiation of OPCs following injury may result in a higher proportion of donor cells forming oligodendrocytes than could be easily obtained from *in vitro* conditions (Karimi-Abdolrezaee et al., 2006). Post-transplant differentiation usually results in a heterogeneous population of cell types, consisting of oligodendrocytes as well as astrocytes and neurons, and occasionally even Schwann cells (McDonald et al., 1999; Akiyama et al., 2001). The extent to which this is a desirable outcome has been debated. If the goal is to strictly target remyelination, then the generation of pure populations of OPCs or oligodendrocytes is emphasized, however, this has rarely, if ever, been achieved. Furthermore, the transplantation of astrocytes and neurons has been linked to positive outcomes, such as the formation of neuronal relays and increasing neuroprotective effects (Erceg et al., 2010; Fischer et al., 2020). Diverse transplant populations may provide a more clinically relevant and combinatorial approach for treating a traumatic injury. One risk of NSC/NPC transplants comes with the propensity of undifferentiated cells for teratoma formation. Without an adequate differentiation protocol, there is a greater chance that some of the transplanted cells will retain their pluripotency and form tumors (Araki et al., 2013). It is thus critical that protocol development includes rigorous validation and documentation of cell lines to minimize this risk.

**Figure 4:**
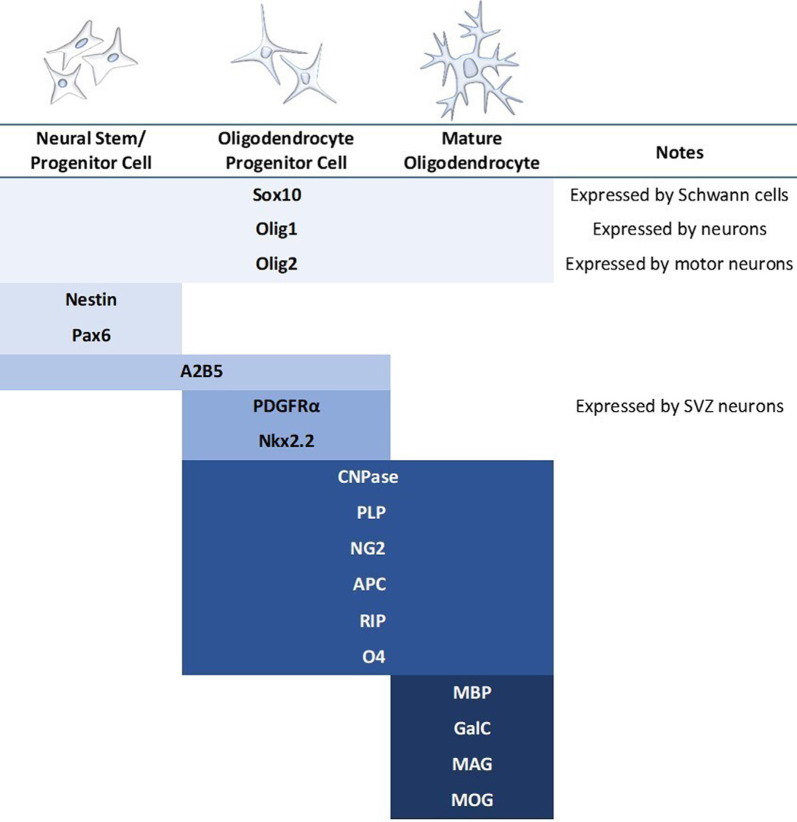
Commonly used markers for the stages of mammalian oligodendrocyte differentiation. Blue bars indicate the stage at which the marker is expressed. A major concern in the literature is the use of only one marker to identify the oligodendrocyte stage when many of these markers express during multiple stages as well as in other cell types. It should also be noted that expression patterns differ based on regional contexts, such as brain vs. spinal cord.

The differentiation and transplantation of OPCs, while one of the most promising areas of SCI research, has also proved to be quite difficult. Early studies went to great efforts to obtain pure OPC populations for transplantation, however, these protocols were lengthy and have been somewhat difficult to replicate (Keirstead et al., 2005; Goldman and Kuypers, 2015). Despite this, there have been several experiments utilizing OPC transplantation in the brain and spinal cord with fair success (Windrem et al., 2004; Hwang et al., 2009; Wang et al., 2013; Kawabata et al., 2016). These studies have reported increased remyelination and spared white matter, synaptogenesis, and locomotor recovery. However, the derivation of OPCs continues to be somewhat elusive. All cells develop along a spectrum, and therefore the “stages” of oligodendrocyte development are less a sequence of discrete subtypes and more a gradual blending from one stage to another. OPCs have been variably defined by a multitude of markers, and while expression levels can be informative about the progression of cell fate specification, there is not an established or reliable point at which a cell is definitively labeled an OPC (Figure 4). Most studies do not have pure OPC populations, but rather “enriched” OPC populations, and thus it is difficult to compare between studies or determine which cell type is providing the most benefit.

Because the role of the mature oligodendrocyte in myelination remains unclear, there are fewer transplantation protocols of fully differentiated oligodendrocytes. A landmark study by Nistor et al. (2005) demonstrated that when transplanted into the spinal cord of a dysmyelinated mouse model, differentiated oligodendrocytes survive, integrate into host tissue, and robustly remyelinate bare axons. Similar results were reported by Liu et al. (2000) when transplanting cultured oligospheres. For the reasons mentioned above, though, it is likely these transplants contained a combination of OPCs and oligodendrocytes at different stages of maturation. Thus, the full contribution of mature oligodendrocytes to myelination has yet to be uncovered.

Though the literature on CRT in TBI is sparse, several recent reviews provide a thorough overview of the application of CRT following traumatic brain injury (Zhou et al., 2019; Zibara et al., 2019). Preclinical trials in rodent models have yielded promising results, and several clinical studies have been proposed in recent years. Reports have documented improved motor and cognitive measures as well as increased neuronal survival following transplantation (Weston and Sun, 2018). However, remyelination has rarely been a focus of cell replacement in TBI to date. Therefore, strong conclusions cannot yet be drawn regarding the effectiveness of oligodendrocytes and remyelination in TBI.

While there is strong therapeutic potential for CRT itself, combinatorial approaches continue to yield the best results in TBI/SCI. Cell transplantation has been paired with RNAi, electrical stimulation, as well as a variety of small molecules to promote neurophysiological recovery (Karimi-Abdolrezaee et al., 2006; Zhang et al., 2012; Jayaprakash et al., 2019). The use of combination therapies has been especially beneficial in developing treatments for chronic SCI. As mentioned above, CRT has proven fairly effective in acute and subacute models of SCI but has largely failed when used in chronic models (Keirstead et al., 2005; Parr et al., 2007; Nishimura et al., 2013). Remarkably, however, recent studies applying multiple strategies have found improvement in chronic SCI animals. Nori et al. (2018) paired OPC transplantation with chondroitinase treatment, reporting increases in remyelination and synaptogenesis giving rise to substantial locomotor improvement. Another team transplanted NPCs with a gamma-secretase inhibitor that promotes neuronal differentiation (Okubo et al., 2018). They also described significant remyelination, functional motor recovery, axon regeneration, and synaptogenesis, the latter likely due to the neural promoter. Given that the majority of SCI cases in the United States are chronic, these studies give hope to previously bleak odds of recovery. As protocols are refined, there is increased potential for combination therapeutics to improve outcomes for patients suffering from traumatic injury.

Additional concerns for the translatability of CRT have expanded the field into large animal models as well. Most TBI/SCI research has been conducted in mice and rats. However, rodents have a greater capacity for endogenous myelination compared to humans, and there is also variability in the size and location of key spinal tracts (Radtke et al., 2007). As such, preclinical trials in non-human primates and other large mammals will be helpful to ensure the safety and efficacy of transplants (Iwanami et al., 2005; Goldman, 2018).

While the large animal literature utilizing CRT for remyelination after SCI is still in its infancy, several key articles should be noted. Pig, dog, and primate models have examined the role of myelination in cell replacement strategies (McMahill et al., 2015; Kim et al., 2018; Rosenzweig et al., 2018). Pig and primate models are commonly used for preclinical trials (Iwanami et al., 2005; Santamaría et al., 2018). Naturally injured dog models are less prevalent but have served as an important bridge between experimentally injured animals and human cases (Granger et al., 2012). Testing the efficacy of treatments in large mammals decreases the risk of clinical trial failure, as differences in smaller animal models may confound the translatability of findings. To date, these models often report various measures of improvement in animals that receive cell transplants, ranging from functional recovery to electrophysiological and histological outcomes. The success of experiments in large mammals will in turn inform continued work in rodent models as protocols are refined and standardized.

Finally, besides TBI/SCI models, many of the studies discussed here also make use of murine models of demyelinating diseases. However, there are some important caveats in comparing the utility of CRT in CNS trauma and in disease. Researchers and clinicians have been optimistic in moving towards a goal of autologous transplants for cases of TBI/SCI, as this would lessen concerns for immune rejection of cells (Forsberg and Hovatta, 2012). This may not be an option for individuals with heritable demyelinating diseases, as the intrinsic capacity of these cells for remyelination may be impaired (Salewski et al., 2015). CRT for these patients then must either use cells from healthy individuals, raising the chances of immunoreactivity, or apply combination therapy with gene-editing technology. It is therefore of utmost importance that, as clinical practices are developed, the risks of immunosuppression are carefully weighed against patient profiles. Taken together, CRT offers a viable future for the treatment of CNS trauma and disease.

## Human Clinical Trials

There have been very few human clinical studies on either TBI or SCI with remyelination as a major or minor goal. We found no registered studies of neural or oligodendrocyte cell transplantation for TBI in human subjects. For remyelination after TBI, one Russian study (NCT02957123) is exploring whether patients with traumatic injury show functional improvement with intranasal inhalations of bioactive factors, produced by autologous M2 macrophages (Ostanin, 2018). This study is not strictly focused on remyelination but mentions it as a potential mechanism. Also, several newly launched clinical trials seek to evaluate the safety and efficacy of CRT in human TBI using mesenchymal stem cells (Schepici et al., 2020). Mesenchymal stem cells (MSCs) have not been discussed in this review article, however, they can be derived from several sources and differentiate into diverse cell types. There is currently no evidence of MSCs becoming myelinating glial cells *in vivo*, however, and thus the extent to which they contribute to remyelination following injury is not understood. Many of these early-phase clinical trials include white matter preservation as an outcome measure, thus when initial results are reported scientists may be able to glean more information about this relationship.

In the field of SCI, while there are several trials investigating ways to overcome the barrier of myelin breakdown products, there are no human trials specifically looking at endogenous remyelination. Several trials have investigated the potential of exogenous remyelination through CRT, either as a primary or secondary goal (Table 1). These trials have utilized a variety of neural cell types for a wide range of injuries and outcome measures. It is thus difficult to make broad conclusions about these studies, so they will be summarized below.

**Table 1:** Summary of current and previous clinical trials utilizing cell replacement therapy in spinal cord injuries (SCI).

Trial	Cell source	Target population	Current status	Trial identifier
Yonsei University (Korea)	Fetal brain-derived NSCs	Acute and chronic cervical ASIA A/B	Completed Phase I/II 2008	NCT0000879
Geron/Asterias Biotherapeutics “SCiStar” (USA)	ESC-derived OPCs	Acute thoracic ASIA A/B Subacute cervical ASIA A/B	Completed Phase I 2011 Completed Phase I/II 2017	NCT01217008 NCT02302157
Stem Cells, Inc. “Pathway” (USA)	Fetal brain-derived NSCs	Subacute/chronic thoracic ASIA A-C Chronic cervical ASIA B/C	Completed Phase I/II 2015 Completed Phase II 2016	NCT01321333 NCT52163876
Miami Project (USA)	Autologous Schwann cells (sural nerve)	Subacute thoracic ASIA A Chronic cervical/thoracic ASIA A-C	Completed Phase I 2016 Completed Phase I 2019	NCT01739023 NCT02354625
Novagenesis Foundation (Russia)	Autologous NSCs +3D matrix	Acute/subacute/chronic, all levels, ASIA A	Completed Phase I 2018	NCT02326662
Neuroregen Scaffold (China)	NSCs + scaffold	Chronic cervical/thoracic ASIA A	Completed Phase I/II 2020	NCT02688049
Neuralstem (USA)	Fetal spinal cord-derived NSCs	Chronic cervical/thoracic ASIA A	Currently enrolling for Phase II	NCT01772810

The first study occurred over 20 years ago at the University of Florida and involved the transplantation of human fetal spinal cord tissue into patients with chronic post-traumatic syringomyelia at the cervicothoracic junction (Thompson et al., 2001). This study demonstrated safety and feasibility in eight patients but was not advanced to further trials. One of the subsequent studies involves the transplantation of Schwann cells from the peripheral nervous system (Anderson et al., 2017). The remaining trials involve the transplantation of either fetal derived neural stem cells (Shin et al., 2015; StemCells, Inc., 2015; Neuralstem Inc., 2017), embryonic stem cell-derived oligodendrocyte progenitor cells (Chapman and Scala, 2012; Lineage Cell Therapeutics, Inc., 2020), or unclear source (Xiao et al., 2016). All trials injected cells directly into the spinal cord.

These trials had mixed results. The safety trials did not report any significant adverse effects and all reported feasibility of this approach (Boulis and Federici, 2011; Guest et al., 2013; Levi et al., 2018). For those that reported outcomes, they were modest and difficult to interpret. The Korean group reported, “moderate neurological benefit” with 5/19 transplanted patients improving at least one level in ASIA score compared to only one patient in the control group that demonstrated improvement (Shin et al., 2015). The SCiStar trial has also reported significant improvement in hand function in their cervical trial, however, this was in comparison to historical controls only, and a randomized controlled trial is planned (Lineage Cell Therapeutics, Inc., 2020). The Pathway trial, a randomized single-blind controlled trial, was terminated after an interim analysis. While there were some promising improvements observed, especially in sensory function, it was clear that they were not going to be sufficient to meet the primary endpoint (Levi et al., 2019). Thus, while these trials have shown promise, mechanisms remain elusive and the contribution of remyelination is not known.

## Conclusion

In the last decade, there has been a steadily growing body of research highlighting the importance of the myelin sheath. This work began with demyelinating and white matter diseases and has since expanded to include CNS trauma and now developmental and psychiatric diseases. As such, treatments and therapies targeting oligodendrocytes and remyelination may have widespread potential for application. This review article has discussed some of the processes that contribute to oligodendrocyte and myelin damage in injury and disease as well as the strategies currently being utilized to promote recovery. Small molecules and monoclonal antibodies are among the most well-established therapeutics, with many commercial and clinical treatments currently available. Gene therapy and cell transplantation are newer approaches that have grown extensively in recent years. RNAi has proven beneficial for a variety of diseases, and it shows great potential for use as a combination therapy in TBI/SCI. Cell transplantation, especially of oligodendrocytes and their progenitors, improves remyelination in animal models and in some cases has been correlated with functional recovery. Although RNAi and CRT based approaches have led to clinical trials, the success of these trials has largely fallen short of expectations. To improve prospects moving forward, a standardization of molecular techniques, delivery methods, culture systems, cell lines, and differentiation protocols is necessary so that we may focus and fine-tune our methodology. This will be critical in ensuring the replicability of our research and the best outcomes for patients.

## Author Contributions

AP contributed to outlining the review and wrote sections of the manuscript. AH-S wrote the first draft of the manuscript. NP and MB wrote sections of the manuscript. All authors contributed to the article and approved the submitted version.

## Conflict of Interest

The authors declare that the research was conducted in the absence of any commercial or financial relationships that could be construed as a potential conflict of interest.
